# Retraction Note: Melting of Pb Nanocrystals Embedded in Al, Si, and Cu Matrices

**DOI:** 10.1186/s11671-020-03381-2

**Published:** 2020-07-14

**Authors:** Huan Wang, Hongzhi Zhu

**Affiliations:** Chinese Academy of Engineering Physics, P. O. Box 919-71, Mianyang, 621900 People’s Republic of China

**Retraction Note: Nanoscale Res Lett 10, 487 (2015)**

**https://doi.org/10.1186/s11671-015-1196-5**

This article has been retracted by the Editors-in-Chief because the authors do not have ownership of the data they report [1]. An investigation by KU Leuven has concluded that all experiments were performed at KU Leuven, Ghent University and the University of Antwerp, and that these universities own the data. Both authors agree with this retraction.
